# Targeting Biofilms in Chronic Wounds: Emerging Strategies with Antimicrobial Nanocomposites

**DOI:** 10.3390/jfb17060282

**Published:** 2026-06-06

**Authors:** Ingrid D. Guerrero-Rodriguez, Chau M. Nguyen, Kytai T. Nguyen, Luis Soto-Garcia

**Affiliations:** Department of Bioengineering, University of Texas at Arlington, Arlington, TX 76010, USA

**Keywords:** chronic wounds, biofilms, nanomedicine, nanocomposites, metal, photocatalytic, photothermal

## Abstract

Chronic wounds present a significant challenge to healthcare systems globally, affecting approximately 1% of the population and severely impacting their quality of life. Biofilm development occurs in approximately 90% of chronic wounds, contributing to an increased prevalence of polymicrobial infections. Currently, there is a large quantity of antimicrobial topical treatments, dressings, and advanced therapies. However, many of them are hindered by the complex biofilm environment, antibiotic resistance, and/or host tissue toxicity. Furthermore, conventional treatments such as debridement, systemic/local antibiotics, and negative-pressure therapy are often ineffective at eradicating biofilms and fostering optimal healing conditions. Nanomedicine approaches have shown promising potential to address the limitations of current treatments. In this review, we discuss the pathophysiology of chronic wounds, the role of biofilms, microenvironmental changes, current treatments and their limitations, and nanocomposite-based strategies to eradicate biofilms and resolve chronic wounds.

## 1. Introduction

Chronic wounds are a major and growing healthcare challenge with high recurrence and serious impacts on quality of life. A defining barrier to healing is the presence of structured microbial biofilms, which protect embedded bacteria from immune clearance and antimicrobial agents. Because chronic wounds are biologically complex (persistent inflammation, impaired perfusion, protease imbalance, and dysregulated signaling), therapies that only target bacteria or only support tissue repair often fail. Nanocomposites provide a platform to combine antimicrobial and wound-modulating functions, enabling targeted biofilm disruption and improved healing conditions [1,2].

In this review, databases including Scopus, Web of Science, PubMed, Google Scholar, and the UTA library were searched with keywords related to chronic wounds, biofilms, and antimicrobial nanocomposites emphasizing recent studies and experimental biofilm data where possible.

### 1.1. Chronic Wounds

Chronic wounds are commonly defined as wounds that fail to heal within ~3 months and impose substantial clinical and economic burden [3,4]. Wound healing involves a series of interconnected biological events aimed at restoring the integrity of the skin. This repair process involves four overlapping phases: hemostasis, inflammation, proliferation, and remodeling. During hemostasis, platelets are activated and start developing a fibrin clot to control bleeding. Then, pro-inflammatory chemokines and cytokines are secreted to draw immune cells to the site, beginning the inflammatory phase [5,6]. Neutrophils are the first immune cells to arrive, clearing away cell debris, damaged extracellular matrix (ECM) components, and pathogens by releasing oxygen-free radicals and elevated levels of proteases. They typically remain active at the site for 2–5 days. Around the third day, pro-inflammatory cytokines (damage- and pathogen-associated molecular patterns, DAMPs and PAMPs) and interferons induce the recruitment of monocytes and their differentiation into M1 macrophages, whose primary role is the production of pro-inflammatory mediators. Additionally, M1 macrophages engage in phagocytosis by releasing matrix metalloproteinases (MMPs) and elastases, thereby regulating proteolytic activity, and orchestrating the transition from inflammation to proliferation. Once M1 macrophages have cleared the damaged tissue and ingested bacteria, several anti-inflammatory molecules (prostaglandins, glucocorticoids, and interleukins 4 and 10) promote their transition into M2 macrophages, whose primary function is to further release anti-inflammatory molecules such as platelet-derived and transforming beta-1 growth factors (PDGFβ and TGF-β1). These growth factors are crucial in managing inflammation, activating fibroblasts, and promoting their differentiation into myofibroblasts for tissue remodeling. They also stimulate angiogenesis, collagen synthesis, and ECM production, thereby facilitating tissue granulation development and aiding tissue repair [7,8,9].

**Figure 1:**
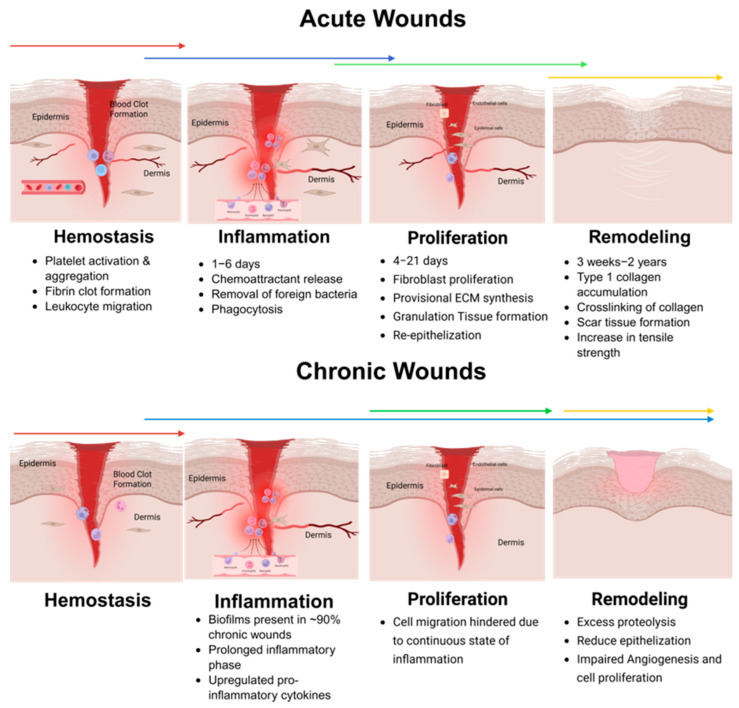
Acute vs. chronic wounds. In chronic wounds, a constant state of inflammation prolongs the inflammatory phase, inhibiting cell migration and proliferation, and upregulating proteases. Hence, reduced epithelization and impaired angiogenesis are observed. Colored arrows represent the time for each phase: red for hemostasis, blue for inflammation, green for proliferation, and yellow for remodeling. Created with BioRender.com.

In contrast, chronic wounds exhibit impaired healing characterized by persistent inflammation (Table 1, Figure 1) [10]. In the United States, approximately 35% of wounds are classified as chronic, including pressure, diabetic, venous, and arterial ulcers (Table 2) [11,12]. These non-healing wounds represent a major public health burden, particularly among elderly individuals and patients with comorbidities such as obesity, diabetes, age, malnutrition, and cardiovascular disease [3,13,14].

While chronic wounds differ in their etiology at the molecular level according to their type, all of them present an immune system dysregulation, which is characterized by overactivity of neutrophils and macrophages, a high presence of T cells, high levels of proteases, high levels of MMPs, senescent cells, and an upregulated presence of proinflammatory cytokines. Furthermore, about 90% of chronic wounds contain polymicrobial biofilms, which aggravate their chronicity by potentially amplifying antibiotic resistance development, as biofilms have one to three orders of magnitude higher resistance to the host’s immune system, antimicrobial agents, and disinfectants [13,21,22,23,24,25,26]. Hence, the degree of colonization by pathogenic bacteria is associated with the level of chronicity observed in chronic wounds [27].

### 1.2. Chronic Wounds Pathogenesis and Biofilms

**Figure 2:**
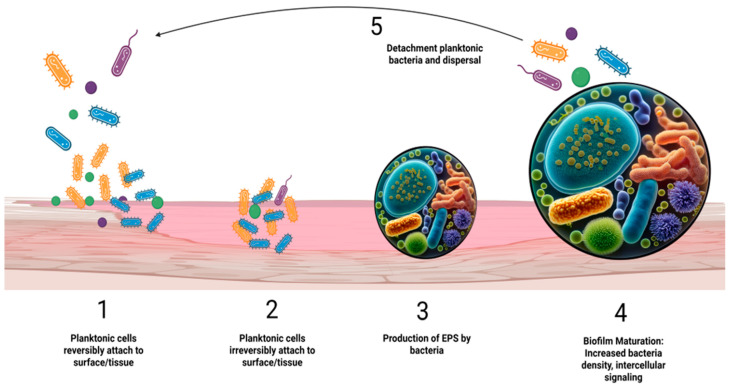
Biofilm formation stages. (1) Planktonic bacteria attach to tissue via electrostatic forces, van der Waals forces and hydrogen bonds, allowing for reversible attachment. (2) Irreversible attachment occurs because of bacterial cell adhesion to the surface through covalent, ionic bonding and dipole-dipole interactions. (3) Bacteria secrete a substrate of extracellular polymeric substances (EPS), which promote biofilm formation. (4) Biofilm develops and matures. (5) After maturation of biofilm, some bacteria detach to become planktonic and disperse, starting the cycle again. Created with BioRender.com. and Adobe.

Chronic wounds are frequently colonized by polymicrobial communities comprising aerobic and anaerobic pathogens, most notably *Staphylococcus aureus* (*S. aureus*) and *Pseudomonas aeruginosa* (*P. aeruginosa*) (Table 3) [28,29,30,31]. These organisms exhibit intrinsic and acquired antibiotic resistance mechanisms that enhance virulence and complicate treatment [29,32]. Biofilms develop when bacteria adhere to tissue, proliferate, and secrete an extracellular polymeric substance (EPS) matrix that provides structural stability and protection. Within this matrix, bacteria exhibit reduced metabolic activity and altered gene expression, increasing tolerance to antimicrobial agents and immune responses. Quorum sensing (QS) further regulates biofilm formation, virulence, and community behavior through density-dependent signaling, reinforcing persistence and recurrence [21,22,29,31,33,34,35,36]. Collectively, biofilm-associated resistance mechanisms—including metabolic dormancy, EPS shielding, and horizontal gene transfer-significantly limit the effectiveness of conventional antibiotic [22,31]. As a result, polymicrobial colonization impairs wound healing and contributes to the chronicity of these wounds (Figure 2) [27].

Biofilms impair host immune function in chronic wounds by reducing macrophage phagocytosis, disrupting ROS signaling, inhibiting neutrophil activity, and limiting effective opsonization through EPS-mediated sequestration [41,42]. These effects sustain a chronic inflammatory state and disrupt key regulatory pathways, leading to reduced growth factor levels (e.g., TGF, VEGF, PDGF, and EGF) and impaired tissue repair [43]. Concurrently, the wound microenvironment becomes progressively dysregulated, characterized by alkaline pH, hypoxia, protease imbalance, reduced cellular proliferation, and fibroblast senescence. These changes collectively reinforce biofilm persistence and create a self-perpetuating cycle in which impaired immunity, tissue degradation, and microbial activity exacerbate one another. Biofilm-associated bacteria further contribute by releasing enzymes and toxins that degrade the extracellular matrix and sustain inflammation. As a result, chronic wounds are marked by persistent inflammation, excessive exudate, and poor-quality granulation tissue, leading to delayed healing and frequent recurrence [29,42,44,45,46,47,48,49,50,51].

### 1.3. Pathophysiological Microenvironmental Changes in Chronic Wounds

Many microenvironmental factors within non-healing wounds undergo changes as chronicity progresses, contributing to biofilm formation and increase in bacterial virulence [52,53]. For instance, Hostacka et al. (2010) [54] showed that biofilm formation by *P. aeruginosa* and *K. pneumoniae* decreases at 37 °C, but increases with higher pH, reaching 139–244% more biofilm at pH 8.5. Similarly, Kim et al. (2020) [55] found that elevated oxidative stress in diabetic wounds reduces microbial diversity and promotes colonization by biofilm-forming pathogens. Conversely, biofilms further alter the wound microenvironment: Thaarup et al. (2023) [56] demonstrated that biofilms shift conditions toward alkalinity (pH ≥ 8.0) and lower oxygen levels due to bacterial consumption. When treatment reduced the number of colony-forming units (CFUs), pH decreased, and oxygen levels increased [50]. Overall, changes in pH, oxygen tension, and ROS create a self-reinforcing cycle that promotes biofilm formation and impairs wound healing.

#### 1.3.1. pH

**Figure 3:**
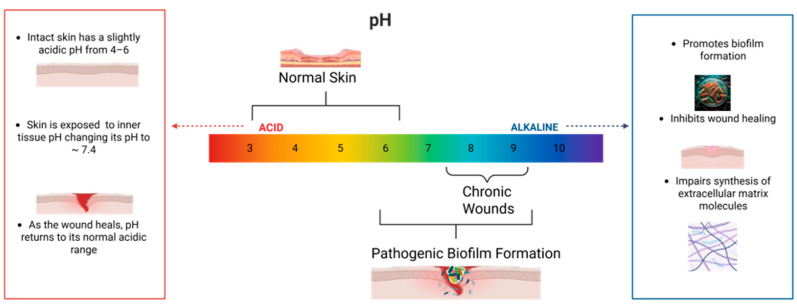
pH changes acute wounds vs. chronic wounds. Wound pH oscillates from acidic to alkaline. Intact skin has a pH of 4–6; after an injury, the skin pH increases due to exposure to tissue pH, and as healing progresses, the pH returns to its baseline acidic level. In chronic wounds, the pH becomes increasingly alkaline due to chronic inflammatory state and bacteria. Created with Biorender.com.

During wound healing, pH regulates key processes including infection control, oxygen release, protease activity, and antimicrobial function [57]. Acute wounds exhibit dynamic pH changes, ultimately returning to an acidic environment that supports fibroblast activity and limits bacterial growth. In contrast, chronic wounds become alkaline (pH 7.15–8.9) due to disrupted biochemical processes, promoting catabolism and biofilm formation (Figure 3) [58]. Consistently, higher pH levels (7.5–8.5) enhance biofilm production, whereas acidic conditions inhibit it [45]. Overall, acidic pH favors tissue regeneration and microbial control, while alkaline pH impairs healing and promotes biofilm development, making pH modulation a critical strategy for managing chronic wounds.

#### 1.3.2. Tissue Oxygen

Oxygen is a critical regulator of wound healing, supporting infection control and key cellular processes such as re-epithelialization, fibroblast proliferation, collagen synthesis, and angiogenesis [59,60]. Following injury, vascular disruption reduces oxygen levels, leading to hypoxia, which initially promotes growth factor-mediated cell proliferation and angiogenesis but requires restoration of vascular supply for proper healing [9,26,61]. Tissue oxygen tension (PtO_2_) declines early and gradually recovers during healing; however, cell function is highly oxygen-dependent. For example, neutrophil antibacterial activity decreases with PtO_2_ below 40 mmHg, while fibroblast collagen production requires 30–40 mmHg [46,62]. Chronic wounds exhibit sustained hypoxia (PtO_2_ 5–20 mmHg) due to impaired perfusion and persistent inflammation, favoring bacterial growth and tissue necrosis [61,63,64]. Thus, restoring oxygen balance is essential for disrupting biofilms and enabling effective wound repair.

#### 1.3.3. Reactive Oxygen Species (ROS) in Wound Healing

Endogenous reactive oxygen species (ROS) are oxygen-derived intermediates, including free radicals such as superoxide (O_2_^−^) and hydroxyl (OH), and non-radicals such as hydrogen peroxide (H_2_O_2_) [64]. ROS contribute to wound healing by eliminating pathogens through oxidative damage (e.g., lipid peroxidation and DNA damage) and by activating immune and cellular responses, including autophagy, neutrophil extracellular trap formation, and T-cell signaling [65]. In addition, ROS act as signaling molecules that regulate key processes such as macrophage activation, angiogenesis (via HIF-1α), and monocyte differentiation through NADPH oxidase pathways [44,66].

**Figure 4:**
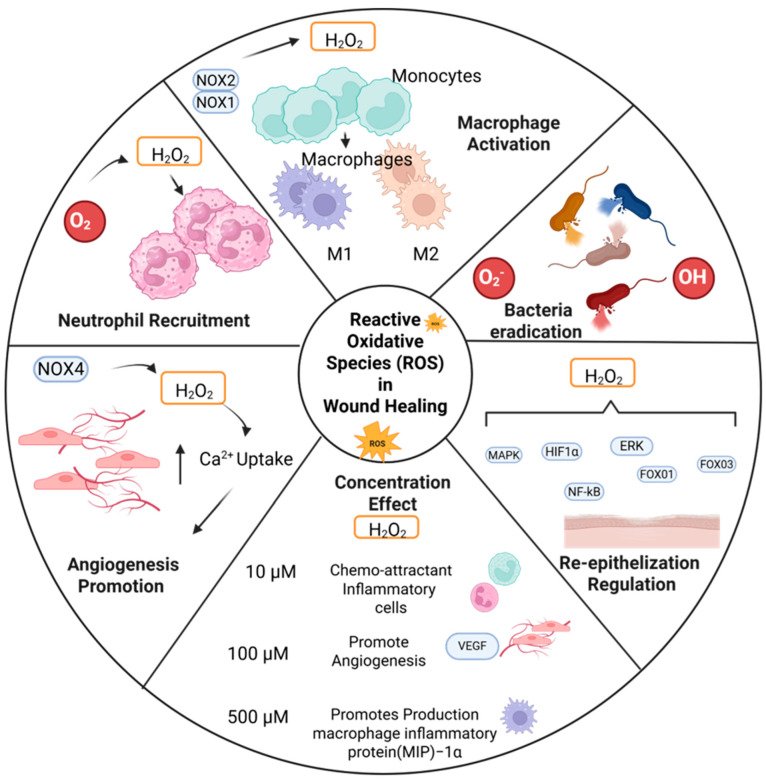
Schematic ROS functions in wound healing. ROS play several roles during wound healing, including immune cell recruitment and activation, bacteria eradication, and the promotion of angiogenesis. ROS function is dependent on their concentration. Created with Biorender.com.

H_2_O_2_ functions as a central secondary messenger, promoting angiogenesis, cell migration, and re-epithelialization in a concentration-dependent manner [66]. Low to moderate levels stimulate fibroblast, endothelial, and keratinocyte proliferation, whereas specific concentrations induce chemotaxis, VEGF signaling, and inflammatory mediator production [64,67,68]. Other ROS, including O_2_^−^ and OH, primarily support antimicrobial defense. However, excessive ROS in chronic wounds lead to sustained oxidative stress, chronic inflammation, and impaired healing (Figure 4) [63].

Exogenous ROS delivery systems, such as H_2_O_2_-releasing bandages and hydrogels, have demonstrated strong antimicrobial activity against wound pathogens [69,70]. Despite these benefits, tight regulation of ROS levels is essential, as high concentrations prolong inflammation and delay healing [44,71]. Overall, balanced ROS production is critical, with levels rising transiently during early inflammation and returning to baseline as healing progresses [66].

Therefore, understanding microenvironmental changes in chronic wounds is fundamental to developing new treatments that effectively address their complexity.

### 1.4. Current Chronic Wound Treatments and Their Limitations

The set of comprehensive approaches to managing diverse types of wounds was established in 2003 by the International Wound Bed Preparation Advisory Board to maximize healing potential; this approach is called T.I.M.E. (Tissue management, Inflammation and infection control, Moisture balance, Epithelial (edge) advancement) (Table 4). First, non-viable tissues are removed through debridement. Then, antibiotics are administered to minimize infection and inflammation. Next, a moisture imbalance is corrected by applying dressings. Lastly, therapies such as growth factors are added to promote epithelization [72,73]. The current standard of care for non-healing infected wounds follows the T.I.M.E approach, with surgical, autolytic/enzymatic, or chemical debridement to remove non-viable necrotic tissue and eradicate biofilms, followed by the application of topical and systemic antimicrobials and concluding with the use of dressings to provide a moist environment and limit bacterial growth [14,46,74,75,76,77]. Other conventional treatments for non-healing wounds include systemic and local antibiotics, hyperbaric oxygen therapy, and negative-pressure wound therapy (NPWT) [78].

One of the major limitations of the T.I.M.E. approach in chronic wound management is the inefficacy of debridement methods in eradicating bacterial infection and biofilm. Debridement can push biofilm structures into deeper tissues, and, when used alone, may not be sufficient for biofilm removal, leading to biofilm regeneration from bacteria left behind within 48 h of debridement [48,76]. Furthermore, other drawbacks include widespread bacterial resistance to antibiotics and disinfectants due to the constant use of systemic antibiotics and antimicrobial topicals, contact dermatitis from NPTW, pain, and toxicity from antimicrobial topicals at high doses [46,79]. Hence, the inefficacy of conventional approaches in treating biofilms is a significant disadvantage that contributes to the high failure and recurrence rates in chronic wounds. For example, diabetic ulcers have a recurrence rate of 60%, pressure ulcers 23–40%, and venous ulcers 24–57% [73,80]. Notably, chronic wounds exhibit a higher 5-year mortality rate than colorectal, prostate, and breast cancer, primarily due to the inefficacy of existing treatment modalities [81]. In general, biofilms complicate treatment options due to antimicrobial recalcitrance and recurrence after debridement [48], as well as their high resistance to current antimicrobials [23]. Although more than 70 products have been approved by the FDA for wound management, only 3 products (Becaplermin gel, Dermagraft, and Apligraft) have been approved specifically for chronic wounds [3]. Given the shortage of products specifically targeting chronic wound treatment, novel approaches are needed to disrupt biofilms in chronic wounds and effectively promote wound healing. The unique properties of nanotechnology-based antimicrobial materials make them excellent candidates for developing optimal materials to disrupt biofilms and help address the shortcomings of current treatments [82].

**Table 4:** Components of the T.I.M.E. approach in wound care.

Class	Examples	Advantages	Limitations	References
**Debridement**				
Mechanical	Surgical, ultrasound therapy	Allows the removal of substantial amounts of necrotic tissue.	May not remove all biofilm components, causing reestablishment of biofilm.	[83,84]
Enzymatic	Collagenase Clostridial collagenase ointment (CCO)	Safe, effective in burns, venous, and pressure ulcers.	Cannot be used with silver-derived products or Dakin solution.	[85]
Chemical	Trichloroacetic acid (TCA) (venous leg ulcers)	Reduction of fibrin observed. Less painful.	Potential pain, damage to tissue.	[86]
**Antimicrobials**				
Iodine-based	Povidone iodine	Kills MRSA within 20–30 sec.; effective against biofilms.	Skin irritation, allergic reactions, tissue toxicity, and rare cases of iodine systemic toxicity.	[87,88]
Silver-based	Actisorb Plus 25, Silverton, and Promogran Prisma	Efficient over a broad bacterial species.	Toxicity	[89]
**Dressings**				
Silver dressings	Atrauman.Ag, Acticoat Flex 3&7, MepilexAg, Biatain Ag, and Silverlon	Efficient against gram-negative and gram-positive bacteria.	Ability to control silver loading, biocompatibility.	[89]
Alginate dressings	Algisite, Tegagen, Kaltostat, Sorbsan, Curasorb, and Seasorb	Low cost, biocompatible, biodegradable, creates a moist wound environment.	Not suitable for dry wounds. Can adhere to the wound bed.	[90]
**Growth factors**				
Platelet-derived growth factor (PDGF)	Regranex (Becaplermin)	Helps in the production of granular tissue.	Only for diabetic foot ulcers (DFU).	[91]

## 2. Nanomedicine Approaches to Eradicate Wound Biofilms

Nanomedicine approaches to treat biofilms in chronic wounds have great potential due to their adaptability, tunability, and ability to cross the biofilm barrier [42,92,93,94]. Reducing a material to nanoscale dimensions increases its surface-to-area/volume ratio, thereby enhancing its physicochemical reactivity [95]. These physicochemical properties make nanoparticles (NPs) excellent candidates for promoting wound healing. NPs can be engineered to mimic cellular components and customized to modify the wound environment [96]. Additionally, they can serve as drug delivery systems, aid in wound repair, be tailored to possess antimicrobial properties, disrupt EPS to target biofilms, and induce biofilm dispersion. For instance, Sedighi et al. (2024) [97] and Jing et al. (2024) [98] reviewed different approaches to target biofilms in wound healing using NPs. They concluded that NP-based systems could combat antibiotic-resistant infections, support biofilm management in chronic wounds, and target specific biofilm-forming phases by adapting to the wound environment, thereby promoting healing. Formulating NPs into nanocomposites is a strategy to enhance their therapeutic potential, as discussed in the following section.

### 2.1. Nanocomposites

Nanocomposites are multiphase materials in which one phase consists of nanoscale particles dispersed within a matrix, offering multifunctional capabilities. Nanocomposites have been shown to promote tissue regeneration, help control infections, and enhance therapeutic outcomes; they can also be engineered to improve mechanical properties, thermal stability, permeability, and electrical conductivity [99,100,101]. Moreover, nanocomposites have been demonstrated to enhance antimicrobial activity and are more effective than NPs alone [1]. They can be engineered to exhibit high selectivity towards bacterial cells and cross the biofilm’s extracellular polymeric matrix [102], making them excellent candidates as antimicrobials against chronic wound biofilms and drug-resistant bacteria. For instance, Luo et al. (2023) [103] developed a nanocomposite of cerium oxide nanoparticles embedded in poly(2-hydroxyethyl methacrylate)-chitosan hydrogels, which were applied as wound dressings. Their results showed that the nanocomposite reduced *S. aureus* biofilms by about 85% and *E. coli* biofilms by 100% in vitro, promoting faster wound closure and complete healing within 14 days in vivo [103]. Another example is the polydopamine NPs/gelatin oxidized dextran nanocomposite developed by Zhang et al. (2025) [104] for diabetic wounds. Their results demonstrated a 99% reduction in bacterial viability for *S. aureus*, *E. coli*, and MRSA [104]. Hence, several studies have proven that combining nanoparticles within a matrix enables the creation of materials with tailored functionalities, resulting in synergistic enhancement of their antimicrobial properties compared to single materials, making them optimal for multiple applications.

### 2.2. Antimicrobial Mechanism of Nanocomposites Against Chronic Wound Biofilms

Antimicrobial nanocomposites eliminate biofilms and planktonic bacteria through several distinct biological mechanisms, including the generation of ROS and oxidative stress, dispersion or disruption of the EPS matrix, physical damage to bacterial cell membranes, release of metal ions, inhibition of quorum-sensing pathways, and a range of synergistic or stimuli-responsive actions that enhance antibacterial performance (Table 5) [105]. In the following sections, we will discuss nanocomposites by mechanistic category.

#### 2.2.1. ROS-Generating Nanocomposites

**Figure 5:**
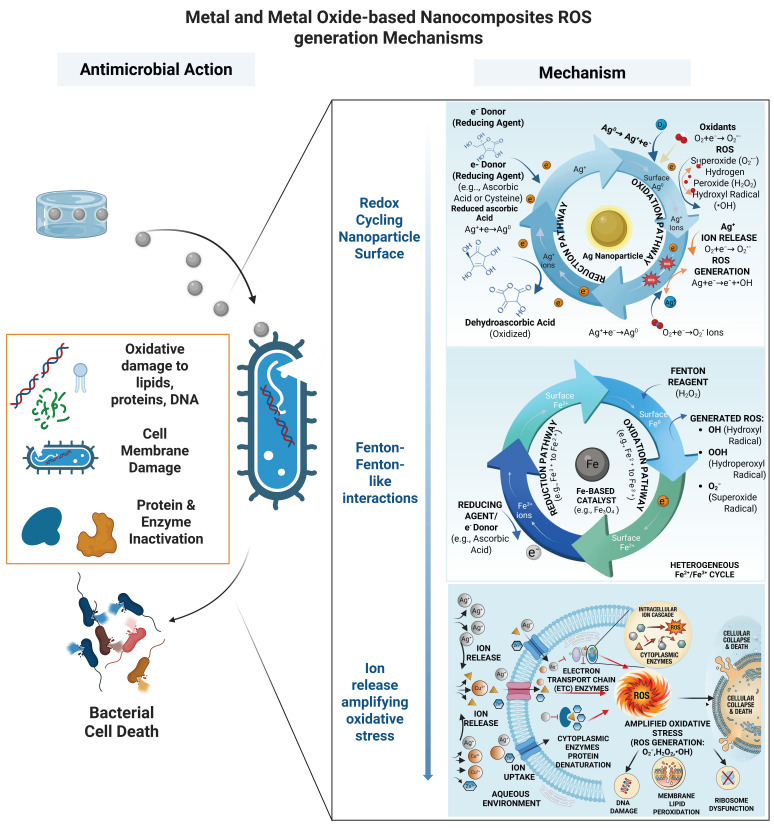
Metal and metal-oxide based nanocomposites mechanism of action. The antimicrobial mechanism of metal and metal oxide materials is based in redox cycling, ion release, and Fenton-Fenton-like interactions. Created with Biorender.com.

The generation of reactive oxygen species (ROS) is a widely employed strategy for disrupting bacterial biofilms in chronic wounds. This approach primarily involves surpassing the bacterial antioxidant capacity, leading to severe oxidative stress that damages both the biofilm extracellular matrix and the membranes of embedded bacterial cells. The resulting effects include ion leakage, membrane depolarization, and compromised structural integrity of bacterial cells. Additionally, certain ROS, such as H_2_O_2_, can penetrate bacterial cells and participate in Fenton-type reactions, producing hydroxyl radicals that damage DNA, disrupt Fe-sulfur cluster-containing enzymes, and impair bacterial respiratory pathways (Figure 5) [105].

Nanocomposites that generate ROS disrupt the biofilm matrix by targeting key extracellular polymeric substance (EPS) components, including polysaccharides, proteins, and extracellular DNA. This process results in matrix fragmentation, increased porosity, and enhanced susceptibility to immune clearance and to delivered antimicrobial agents. Moreover, metal and metal-oxide nanoparticles or photocatalytic materials capable of generating ROS are used to develop nanocomposites that create localized oxidative microenvironments that accelerate EPS degradation and bacterial killing, enabling deeper penetration and disruption of mature biofilms [97,105].

##### Metal and Metal-Oxide-Based Nanocomposites

Metals such as titanium, iron, silver, copper, zinc, cerium, and their oxide-based nanoparticles have been widely explored for biofilm management due to their intrinsic antimicrobial properties and their ability to penetrate biofilms more effectively than bulk materials. Their nanoscale dimensions, high surface area, and tunable surface charge facilitate close interactions with bacterial cells and the EPS matrix [2,113,114,115]. The antibacterial activity of metallic nanoparticles generally arises from three interconnected mechanisms: a) oxidative stress through the production of ROS, b) physical disruption of the bacterial cell wall and membrane, and c) interference with intracellular structures and protein function through the release of metal ions [116,117]. For example, silver and copper-based nanocomposites generate ROS primarily through surface redox cycling, the process in which the redox-active surface of nanoparticles releases electrons that reduce molecular oxygen to superoxide (O_2_^−^). Then, superoxide is further converted into H_2_O_2_ and hydroxyl radicals (OH). In parallel, released metal ions (e.g., Ag^+^, Cu^2+^, and Zn^2+^) disrupt bacterial redox homeostasis by inhibiting antioxidant enzymes such as catalase and superoxide dismutase, damaging Fe-S cluster proteins, and increasing intracellular ROS leakage from respiration [118].

These combined effects of oxidative and ionic stresses inhibit the development of antimicrobial resistance. Incorporating metal or metal oxide nanoparticles into matrices to create nanocomposites enhances their efficacy by increasing stability, regulating ion release, and introducing additional bioactive properties. For instance, Ghosh et al. (2022) [119] developed a quaternary chitosan derivative silver chloride nanocomposite (QAm1Agn) that achieved log reductions of 6.2, 5.0, and 4.5 against *P. aeruginosa*, *A. baumannii*, and MRSA, respectively, and also reduced polymicrobial biofilms [119]. Similarly, Rasool et al. (2022) [120] developed a functionalized silica-ceria nanocomposite that inhibited *S. aureus* (99.9%) and *E. coli* (81%), prevented biofilm formation by 80%, and disrupted established biofilms by 77% [120].

Despite their strong antibiofilm activity, metal and metal-oxide nanocomposites can exhibit dose-dependent cytotoxicity when used alone at high concentrations [121]. Therefore, integrating them into biocompatible matrices or combining them with complementary therapeutic strategies is essential to balance antimicrobial efficacy with safety to support their clinical translation [122].

##### Photocatalytic-Visible Light Responsive Systems

**Figure 6:**
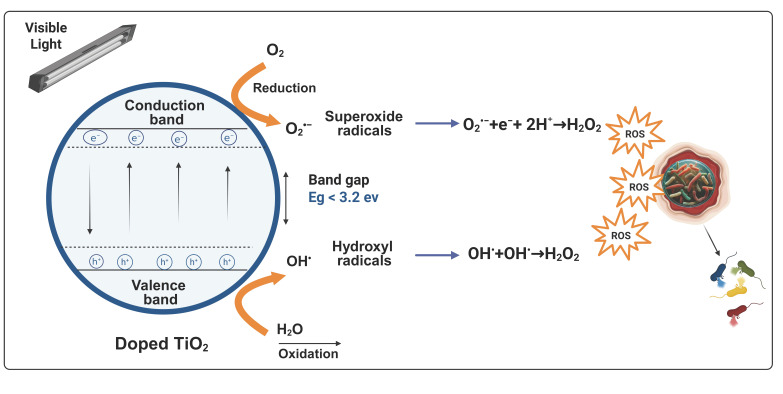
Photocatalytic antimicrobial mechanism of doped TiO_2_. Polydopamine (PDA)-doped TiO_2_ can absorb light in the visible region. After light irradiation, photogenerated electrons from conduction band recombine with holes in the valence band of the reduction-photocatalyst (TiO_2_). Then, PDA acts as an electron acceptor transferring photogenerated electrons to PDA surface and inhibiting the recombination of electron-hole pairs, increasing uptake of molecules to reactive site and enhancing production of ROS. Created with Biorender.com.

Photocatalytic nanocomposites are a major class of ROS-generating materials used to disrupt biofilms in chronic wounds. When semiconductor nanoparticles absorb light energy (photons) equal or greater than their bandgap energy (E_g_), electrons in the valence band (VB) become excited and transition to the conduction band (CB), leaving behind positively charged holes (h^+^). These electron-hole pairs drive redox reactions at the nanoparticle surface: photogenerated electrons reduce molecular oxygen (O_2_) to superoxide radicals (O_2_^−^), while holes oxidize water (H_2_O) or hydroxide ions (OH^−^) to hydroxyl radicals(·OH) (Figure 6). Additional ROS, including singlet oxygen (^1^O_2_) and H_2_O_2_, further amplify oxidative stress. Together, these species damage bacterial membranes, proteins, nucleic acids, and the extracellular polymeric substances that stabilize biofilms. Nanocomposites enhance photocatalytic performance by combining multiple materials to broaden light absorption, suppress electron-hole recombination, and increase surface area for ROS-generating reactions [123,124].

Photocatalytic nanocomposites are considered safer and more effective for chronic wound treatment. These materials generate controlled, surface-localized ROS only when exposed to a specific activating wavelength. This light-dependent activation limits unnecessary ROS production. It also reduces oxidative stress in surrounding healthy tissue and minimizes the cytotoxicity often associated with metal nanoparticles that continuously release ions [125,126].

An example of a photocatalytic nanocomposite is the graphene oxide-based nanocomposite developed by Prakash et al. (2022) [127]. This material was tested against biofilms of *E. coli*, *S. aureus*, *P. aeruginosa*, and *E. faecalis*. Their results indicated that the GO/TiO_2_(V–N) nanocomposite exhibited enhanced antibacterial activity and effectively inhibited EPS production and biofilm formation across all four bacterial strains [127]. Similarly, Prema et al. (2021) [128] synthesized a GO/Zn (Cu)O nanocomposite. This compound demonstrated a synergistic effect upon light exposure, amplifying antimicrobial and antibiofilm activity. The effect arises from the combined photocatalytic properties of the metal oxide nanoparticles and graphene oxide (GO), which acts as an electron acceptor and transporter. It reduces electron-hole recombination and increases ROS production. This demonstrates that incorporating Zn and Cu O NPs into the GO matrix, combined with the photocatalytic effect, produces a synergistic antimicrobial response that surpasses that of the nanoparticles alone or in the dark [128].

In addition to their antimicrobial properties, photocatalytic nanocomposites can promote wound healing when exposed to light in the 405–904 nm range. When photocatalytic nanocomposites are exposed to light irradiation within these wavelengths, key cellular processes are stimulated, including DNA/RNA synthesis and protein production [129]. For example, blue light (BL) wavelengths (400–470 nm) promote angiogenesis, collagen synthesis, fibroblast migration, and proliferation. They also reduce inflammation and stimulate nitric oxide (NO) production, a vasodilator that can increase blood flow. Furthermore, BL can kill bacteria without the need for photosensitizers. It activates bacteria’s endogenous photosensitizers, mainly porphyrins, whose maximum absorption is at 405 nm [130]. For instance, BL demonstrated having a bactericidal effect against MRSA, causing damage to the bacterial cell membrane, leading to leakage of cellular components and complete lysis [131]. BL was also tested in 34 clinical pathogen isolates. The findings indicated that it can be used as an antimicrobial agent against a wide range of planktonic bacteria and mature biofilms from clinical pathogens [130]. Nevertheless, a significant drawback of BL is its limited depth of penetration, which can result in partial eradication of bacteria within biofilms. This issue can be addressed by combining BL therapy with NIR, as NIR has deeper tissue penetration. This combination produces a synergistic effect that disrupts biofilms in chronic wounds.

#### 2.2.2. EPS Matrix Dispersal/Degradation Nanocomposites

**Figure 7:**
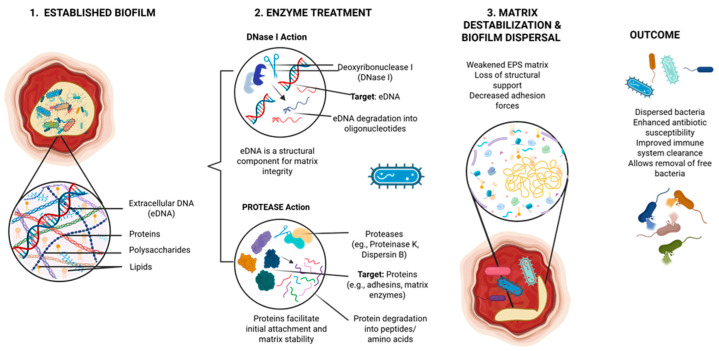
Enzymatic EPS matrix dispersal and degradation mechanism. DNase I targets eDNA, the structural component of EPS matrix. Proteases target EPS proteins like adhesins and matrix enzymes. Created with Biorender.com.

The EPS matrix of chronic wound biofilms represents one of the most significant challenges for antimicrobial agents. EPS is mainly composed of polysaccharides, proteins, nucleic acids, and lipids that form a protective barrier, severely limiting the penetration of antimicrobial agents and promoting their inactivation or destruction through electrostatic interactions or enzymatic degradation [132]. Nanocomposites targeting the EPS use dispersion mechanisms to disrupt biofilms in chronic wounds by breaking down the EPS matrix [97]. Dispersion is when bacteria encased within biofilm are released through passive or active mechanisms, returning to their planktonic state. Passive dispersion relies on triggers mediated by physical external forces that directly release cells from the biofilm. In contrast, active dispersion mechanisms are initiated by antibiofilm stimuli such as enzymes that target EPS matrix components. The main dispersion strategies in nanocomposites are active dispersal using enzyme-functionalized carriers (proteases or DNase), and NO delivery systems (Figure 7) [133,134].

##### Protease-Functionalized Nanocomposites

Protease-functionalized materials use enzymatic degradation of the cellular EPS to physically free embedded bacteria and enable antibiotic access. Several proteinases have been observed to possess biofilm-dispersal capabilities, including proteinase K, which has shown bacterial dispersal against 24 and 48 h *S. aureus* biofilms and *E. coli* biofilms.

Other proteinases include trypsin, pepsin, and carvacrol which can disperse *P. aeruginosa* and *E. faecalis* biofilms [135]. The main mechanism by which proteases promote biofilm dispersal in chronic wounds is enzymatic hydrolysis of protein components within EPS and cleavage of adhesins [134]. For example, Weldrick et al. (2019) [136] developed a protease-functionalized nanocomposite using a Carbopol Aqua SF1 nanogel to immobilize Alcalase 2.4 L FG, a serine endopeptidase. The nanocomposite achieved a 6-fold reduction in biofilm mass for *P. aeruginosa*, *S. epidermis*, *S. aureus*, and *E. coli*, and a 3-log reduction in viable forming cells, confirming the ability of protease-functionalized nanocarriers to disrupt and clear bacterial biofilms [136]. In another proteolytic nanocomposite formulation designed by Azhdari et al. (2025) [137], serratiopeptidase (STP) was encapsulated within a niosomal formulation and incorporated into a nanocellulose gel. The results showed the system had the capability to enhance STP enzymatic activity and stability and allow for STP extended release. However, results showed only ~50% biofilm inhibition against *S. aureus* and *P. aeruginosa*, whereas free STP demonstrated ~60% biofilm inhibition [137].

##### DNase I-Functionalized Nanocomposites

Deoxyribonuclease I (DNase I) is frequently used to degrade the extracellular DNA (eDNA) component of biofilms via hydrolysis. eDNA is a functional and structural component of the EPS matrix in bacterial biofilms; its primary functions are to facilitate initial adhesion, provide resistance to environmental stress, and maintain structural integrity [138]. Incorporating DNase into nanocomposites increases its stability and protects it from proteolysis, allowing dispersion of the EPS matrix while enhancing antibiotic susceptibility, improving immune system clearance, and producing an overall enhanced antimicrobial effect [139,140]. For example, Patel et al. (2019) [141] incorporated DNase-I into a chitosan gel loaded with solid lipid silver sulfadiazine (SS) nanoparticles. The results showed that incorporating DNase-I into nanoparticles and a chitosan gel resulted in higher inhibition (96.8%), while the SS/DNase I combination achieved 82.9%, indicating enhanced antimicrobial activity of the nanocomposite [141].

While enzyme-embedded nanocomposites have been shown to enhance biofilm inhibition and disruption, due to the polymicrobial nature of biofilms in chronic wounds and the changes in EPS matrix composition across species, creating effective systems would require a combination of mechanisms [109].

#### 2.2.3. Contact-Killing/Membrane Disruption Nanocomposites

Contact-killing nanocomposites disrupt biofilm-embedded bacteria through direct physical or electrostatic interactions with the cell membrane. Unlike traditional antibiotics, these nanocomposites act on contact, providing localized killing of bacteria in biofilms and preventing the development of antimicrobial resistance.

##### Electrostatic Interactions

Electrostatic interactions are among the most fundamental and widely exploited mechanisms of contact-killing in antimicrobial nanocomposites. Bacterial cell surfaces are inherently negatively charged due to the teichoic acids of gram-positive bacteria and the lipopolysaccharides of gram-negative bacteria. Nanocomposites designed with positively charged polymers, cationic metal species, and functional groups (e.g., chitosan and metal oxides) can bind strongly to bacterial cell membranes through electrostatic attractions. This destabilizes the membrane by increasing permeability, disrupting the lipid bilayer, and causing leakage of intracellular components [98]. Biofilm’s EPS matrix also has a net negative charge, allowing cationic nanocomposites to penetrate more effectively and kill biofilm-embedded cells [111].

**Figure 8:**
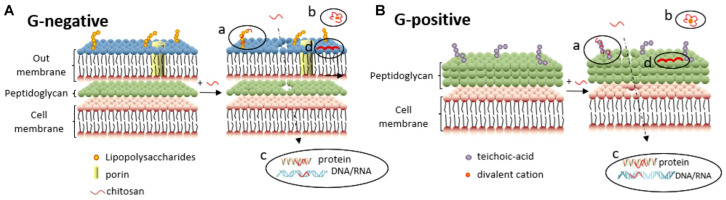

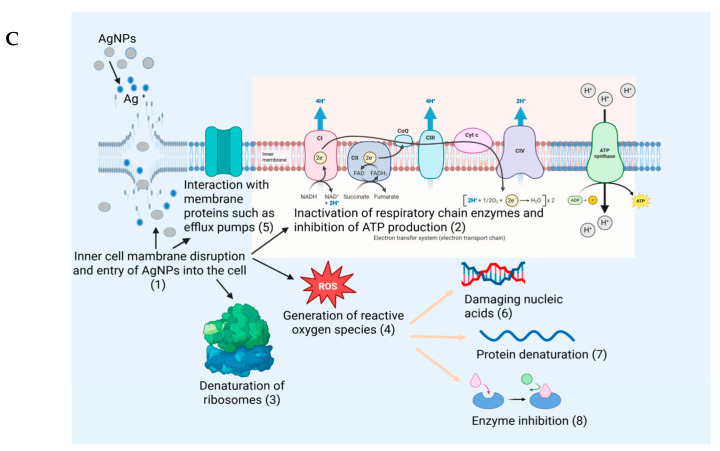
Antimicrobial mechanism of chitosan and silver nanoparticles. (**A**) Chitosan electrostatic interactions with gram-negative bacteria. (**B**) Divalent cations from gram-positive bacteria are chelated by chitosan (Reprinted from Ref. [143]). (**C**) Antimicrobial mechanism of Ag NPs (Reprinted from Ref. [144]).

Nanocomposites that use electrostatic interactions as their primary mechanism of action are more effective against gram-positive bacteria because these bacteria are inherently more negatively charged, thereby attracting and diffusing positively charged nanoparticles more efficiently [110]. The most common materials used to develop nanocomposites with electrostatic interactions are chitosan, silver, metal oxides, and graphene oxide [142]. Chitosan is a cationic polymer that can interact with the anionic surfaces of EPS and bacteria, leading to membrane depolarization and disruption [110,143]. Similarly, silver nanoparticle-based nanocomposites release positively charged ions that bind to negatively charged bacterial surfaces, triggering ROS production, and leading to biofilm disruption and bacterial death (Figure 8) [144].

##### Photothermal Nanocomposites

**Figure 9:**
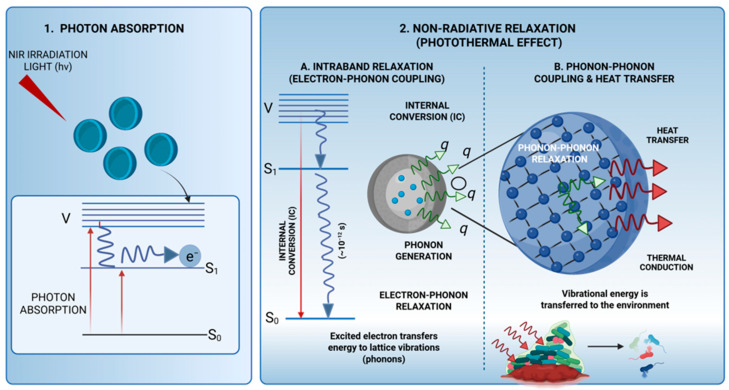
Photothermal antimicrobial mechanism of action. When a photothermal agent absorbs light, the absorbed light energy excites its electrons through localized surface plasmon resonance or electron-hole pair. Once the electrons relax, they transfer energy to the atomic lattice creating phonons (electron-phonon coupling) that rapidly generate heat. This heat is then dissipated to surrounding cooler regions (bacterial cells and environment) increasing the temperature. Created with Biorender.com.

Photothermal (PT) nanocomposites disrupt biofilms and kill bacteria by combining localized heat, structural damage, and synergistic biochemical effects that overcome the natural defenses of biofilm-embedded microbes. PT nanocomposites’ antimicrobial mechanism is based on photothermal therapy (PTT) or the conversion of NIR light into localized heat [145,146]. Upon NIR irradiation, photothermal agents (PTAs) convert absorbed light into thermal energy, thereby elevating local bofilm temperature. This localized heat softens and destabilizes the EPS matrix by inactivating its nucleic acids and proteins, hence increasing its permeability and exposing previously protected bacteria. Then, the bacterial cell membrane is disrupted, and intracellular components are denatured, leading to bacterial cell death (Figure 9) [147,148]. The penetration of NIR light photonic energy through tissues is dependent on the wavelength used, the energy produced, absorption, scattering, the area of irradiance, and pulsing. The infrared (IR) spectral region used in PTT, ranging from 760 to 1400 nm, can penetrate deeply into dermal tissue, with wavelengths between 810 and 830 nm (near-infrared (NIR)) showing the greatest penetration depth (~30–40 mm). Besides the antimicrobial potential of PTT on eradicating biofilm and antibiotic-resistant bacteria in chronic wounds, they can accelerate wound healing by promoting fibroblast proliferation, regulating inflammatory responses, and stimulating angiogenesis—the formation of microvessels [146,149,150].

As a single modality, PTT has major drawbacks, including the need for high doses of photothermal agents (PTAs) and high-power excitation light to achieve the desired antimicrobial effect, which can result in unwanted tissue damage and inflammation, further worsening the chronic wound state. To overcome this PTT limitation, photothermal-responsive nanocomposites have emerged as a promising approach for treating chronic wounds. Hydrogels have a high capacity to absorb large amounts of water, which can help stabilize the temperature within the wound site, thereby avoiding damage to healthy tissue. Incorporating NPs into hydrogels for PTT creates a versatile platform that can significantly enhance PTT effectiveness while further minimizing damage to healthy tissues due to NPs’ ability to localize heat in a specific area when light is absorbed [151,152].

For example, Wang et al. (2023) [153] developed an NIR-II-triggered nanocomposite formed of kanamycin-derived carbon nanodots and nitric oxide donors loaded into dendritic silica-coated three-layer core-shell conversion materials. The results showed peroxynitrite-enhanced MRSA antibiofilm activity, with 95.9% biofilm clearance and a 7.5-fold reduction in EPS. Furthermore, in vivo results showed ~90.7% wound healing in the diabetic wound model [153]. Another example of a photothermal nanocomposite is a multi-functional NIR-triggered injectable hydrogel developed by Du et al. (2023) [154]. The nanocomposite was composed of platinum-modified sodium nitroprusside-porphyrin metal-organic framework with gold nanoparticles. The antimicrobial activity of the PSPG hydrogel was tested against *E. coli*, MRSA, *S. aureus*, *Staphylococcus epidermidis* (*S. epidermidis*), *Salmonella typhimurium* (*S. typhi*), *Klebsiella pneumoniae* (*K. pneumoniae*), and *P. aeruginosa* biofilms. The results indicated that NIR irradiation alone had no effect on disrupting the biofilm; meanwhile, when the nanocomposite (250 μg/mL) was exposed to NIR irradiation (660 and 808 nm), a 99% bacterial inhibition was achieved within 10 min [154]. Furthermore, incorporating materials that enhance photothermal efficiency is crucial when developing nanocomposites for chronic wounds; one such material is polydopamine (PDA), a polymer with strong NIR absorption that enhances photothermal conversion efficiency [155]. An example of a PDA-based nanocomposite used with PTT therapy is a system developed by Gao et al. (2019) [156], in which polydopamine nanoparticles are used in combination with chitosan to create an NIR-responsive system to deliver antibiotics into the wound bed. The results showed a 98.9% bactericidal efficacy against *S. aureus*, higher fibroblast proliferation, thicker epidermis, and more blood vessel formation [156].

While these studies have shown that photothermal responsive nanocomposites are a promising approach for the treatment of chronic wounds, when used as a single treatment, they may not address all the contributing factors to chronic wound pathology. Thus, they may fail to achieve complete eradication of bacteria within the wound environment.

#### 2.2.4. Quorum-Sensing (QS) Inhibition/Metabolic Interference

Biofilms in chronic wounds are regulated by quorum sensing, allowing bacterial cells to communicate via chemical signaling molecules and helping coordinate gene expression of biofilm phenotype when populations are high. This allows bacteria to produce EPS, activate virulence factors, and increase antibiotic resistance [157,158]. Disrupting QS or bacterial metabolic pathways weakens the biofilm’s structural integrity and resilience, making bacteria more susceptible to antimicrobials and immune clearance. Structural modifications of QS signals can disrupt communication and reduce pathogenicity [112].

Nanoparticles designed to disrupt QS inhibitors or interfere with bacterial metabolic pathways can antagonize receptors, disrupt biofilms, and degrade signaling molecules, thereby enhancing biofilm susceptibility to antimicrobial agents. Nanocomposites use multiple mechanisms to suppress QS, including enzymatic inactivation of autoinducers, interference with QS receptors by metallic nanoparticles, and gene-level QS suppression. For example, the Las/RhL/agr QS circuits of *P. aeruginosa* and *S. aureus* are targeted to reduce virulence [159,160]. In other cases, natural phenolics are incorporated into nanocomposites to down-regulate QS AI-2 signaling genes (e.g., luxS, pfs), resulting in reduced EPS and decreased bacterial motility [161,162].

#### 2.2.5. Stimuli-Responsive Activation

Stimuli-responsive nanocomposites are designed to change or enable targeted therapeutic actions in response to specific exogenous or intracellular microenvironmental stimuli such as pH, enzymes, redox potential, temperature, magnetism, light, and electricity. By integrating these stimuli-responsive mechanisms, nanocomposites can precisely target bacteria within biofilms [163,164], enhance tissue penetration, and reduce the development of antimicrobial resistance [165], making them a promising approach to overcoming the limitations of conventional chronic wound treatment.

In the context of chronic wounds, stimuli-responsive nano systems can be engineered to respond to pH, enzymes, redox gradients, temperature, magnetism, and light (photothermal and photocatalytic).

##### pH-Responsive: Nanocomposites

Chronic wound biofilms create an alkaline microenvironment that differs from that of healthy skin [45,58,166]. pH -responsive systems exploit this gradient to trigger antimicrobial peptide release [167], activate catalytic nanozymes, promote charge switching to enhance bacterial membrane binding, and disrupt EPS structure.

##### Magnetic-Responsive Nanocomposites

Magnetic-responsive nanocomposites are highly beneficial for treating chronic wound biofilms because they provide on-demand, targeted, non-invasive therapy. They combat biofilms through a multimodal mechanism combining mechanical disruption, magnetothermal hyperthermia, catalytic ROS generation, and targeted drug delivery. When exposed to an external alternating magnetic field (AFM), magnetic nanoparticles generate localized heat and mechanical motion, disrupting the protective EPS matrix and allowing antimicrobial agents to penetrate deep into the biofilm [168]. Tran et al. (2023) [169] developed multifunctional Fe_3_O_4_ nanoparticles filled with polydopamine hollow rods (Fe_3_O_4_ @PDA) for the treatment of *S. aureus* biofilms. Fe_3_O_4_ @PDA demonstrated the ability to physically disrupt *S. aureus* biofilms under a rotational magnetic field, reducing biofilm biomass by up to 40% after 20 min [169]. In another study, supermagnetic IONPs/AG NPs were developed by Eghbalifam et al. (2023) [170]. Oxidative stress induced by IONPs and their ability to bind to the bacterial cell surface were shown to inhibit bacterial adherence factors. IONPs superparamagnetic properties increased the antimicrobial activity, with *P. aeruginosa* showing the highest sensitivity to the antimicrobial nanocomposite, whereas MRSA showed the lowest. The combination of the mechanism of action from IONPs/Ag NPs when exposed to EMF increased biofilm inhibition from 15% to 40%, showing the advantages of using multiple mechanisms simultaneously to eradicate biofilms [170].

#### 2.2.6. Multimodal Synergistic Nanocomposites

Multimodal nanocomposites that integrate two or more antimicrobial modalities have emerged as a powerful platform for combating biofilms in chronic wounds more effectively. Combining multiple mechanisms to overcome chronic wound biofilms and bacterial resistance can result in systems that enhance antimicrobial and therapeutic effects while simultaneously reducing secondary effects. For example, photothermal systems produce localized hyperthermia to disrupt bacterial membranes and biofilm matrices; photocatalytic components produce ROS that oxidatively damage cellular components. When combining these effects into single nanoplatforms, effective bacterial eradication can often be achieved at a lower temperature and lower doses than with any single modality alone, reducing collateral damage to host tissues and limiting systemic toxicity, which have the potential to target bacterial infections, disrupt biofilm, modulate wound microenvironment, and actively promote wound healing [146].

##### Photo-Catalytic/Dynamic and Photothermal Nanocomposites

Combining photothermal with photocatalytic responsive systems can lead to synergistic effects, enhancing the antimicrobial efficacy and reducing the risk of side effects and the development of antibiotic resistance [145]. Photothermal therapy generates heat in a spatiotemporally controllable manner to eradicate bacteria, and, when combined with photocatalytic or photodynamic modalities, can accelerate intracellular permeation [147]. This synergy arises because heat, light-activated catalysis, and ROS generation reinforce each other at every level of biofilm disruption. Photocatalytic nanomaterials generate ROS upon light excitation, but their catalytic efficiency depends on temperature [171]. The photothermal effect boosts photocatalysis through local hyperthermia (45–55 °C), which increases electron mobility in semiconductor materials, reducing electron-hole recombination, allowing for more electrons to participate in reactions to form ROS, and accelerating surface redox reactions. Furthermore, heat denatures EPS proteins. ROS generated by catalysis have very short diffusion distances; the photothermal effect can allow ROS to penetrate deeper, reach sessile bacteria, and oxidize intracellular targets [172]. For example, Cheng et al. (2023) [173] used the MXene@Zn-MOF nanocomposite, which exhibited photothermal and photocatalytic properties, to provide antibiofilm therapy for infected wounds. The combined mechanism consisted of MXene’s strong photothermal conversion and photocatalytic ROS formation by ZN^2+^ ions. Results showed that the nanocomposite disrupted the EPS matrix and allowed ROS to reach deeper tissues [173].

**Figure 10:**
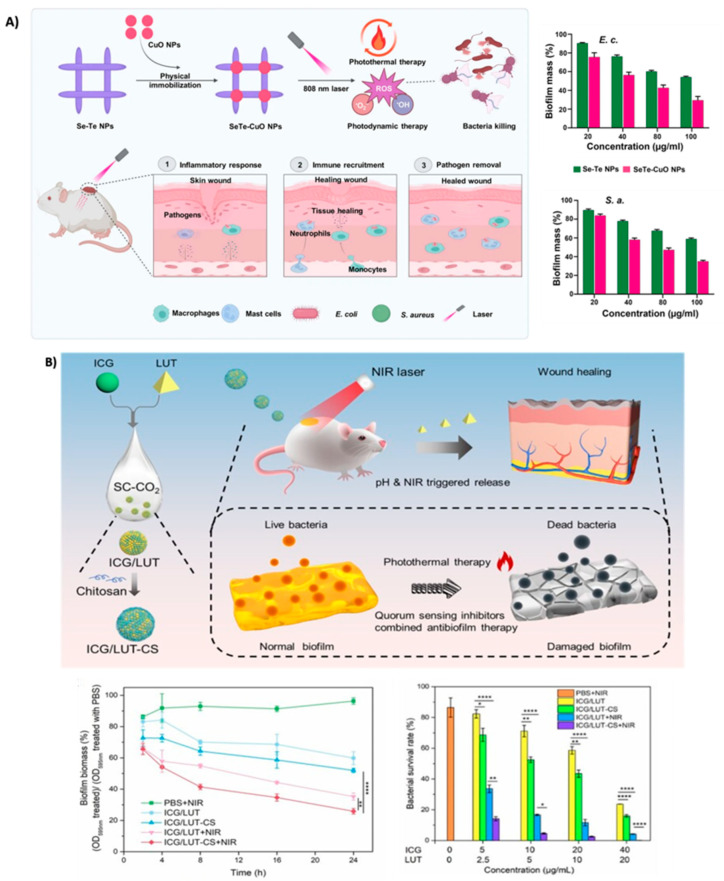
Multimodal synergistic nanocomposites. (**A**) Photocatalytic and ROS-generation multimodal SeTe-CuO nanocomposite with their antibiofilm activity analysis (Reprinted from Ref. [174]). (**B**) Synergistic photothermal and quorum-sensing inhibition nanocomposite. *S. aureus* biofilm biomass reduction and bacterial survival rate (Reprinted from Ref. [175]) The difference among groups in the graphs is represented by not statistically significant (ns), or *p*-values < 0.05 (*), *p* < 0.01 (**), and *p* < 0.001 (****).

In another example, the combination of photothermal and photodynamic modalities used by Ullah et al. (2024) [174] in a selenium-tellurium-doped copper oxide nanoparticles-based nanocomposite demonstrated a synergistic effect that enhanced the antibacterial and antibiofilm activity against *S. aureus* and *E. coli* biofilms, showing a promising alternative for the treatment of drug-resistant bacteria (Figure 10A) [174].

##### Photothermal and Quorum-Sensing Inhibition Nanocomposites

The integration of photothermal therapy with quorum-sensing inhibition represents a strategy that merges physical destruction through heat with molecular disruption by blocking bacterial communication. This approach enables the application of low-temperature (40–45 °C) photothermal therapy, minimizing tissue damage. For instance, the development of a chitosan-indocyanine green/luteolin nanocomposite achieved a 99.9% biofilm inhibition in vitro and 89% wound closure in vivo (Figure 10B) [175].

Overall, while nanocomposites employing individual antimicrobial mechanism demonstrated clear capacity to target and reduce biofilm-associated bacteria, multifunctional nanocomposites proved more effective in addressing the complex, multilayered architecture of chronic wounds biofilms. By combining complementary actions within a single platform, these advanced systems achieved more efficient biofilm disruption and enhanced antibacterial performance compared to single-mechanism counterparts. Table 6 provides a consolidated overview of these nanocomposites organized by their antimicrobial mechanism.

### 2.3. Critical Analysis of Nanocomposite Platforms

While diverse antimicrobial nanocomposite platforms have been developed to target chronic wound biofilms, their effectiveness is highly dependent on wound-specific constraints, including hypoxia, polymicrobial composition, and microenvironmental instability. A comparative evaluation highlights that no single mechanism is universally optimal, and each platform exhibits distinct advantages and limitations that define its translational potential.

#### 2.3.1. ROS-Generating Systems

ROS-generating nanocomposites offer broad-spectrum antimicrobial activity and the ability to penetrate and degrade the EPS matrix through oxidative stress. This makes them particularly effective against mature biofilms. However, their performance is strongly influenced by moisture and oxygen availability as well as local antioxidant defenses. In hypoxic chronic wounds—where oxygen tension is often limited—ROS-mediated killing can be significantly reduced. In addition, uncontrolled ROS generation increases the risk of host tissue damage, limiting safe dosing thresholds.

#### 2.3.2. Contact-Killing Platforms

Contact-killing nanocomposites (e.g., cationic polymers and metal-based surfaces) operate independently of oxygen availability and induce rapid bacterial membrane disruption. These systems are effective against dormant or metabolically inactive bacteria, a key advantage in chronic wounds. However, their action is typically localized to direct contact surfaces, limiting penetration into deeper biofilm layers. Furthermore, strong membrane-disruptive interactions can result in cytotoxicity to host cells if not carefully engineered.

#### 2.3.3. EPS-Disrupting Systems

EPS-targeting nanocomposites directly address the primary structural barrier of biofilms, enhancing permeability and improving the efficacy of co-delivered antimicrobials. However, their effectiveness is highly dependent on biofilm composition, which varies across species and wound types. Enzyme instability and rapid degradation in protease-rich chronic wound environments also limit their durability.

#### 2.3.4. Quorum-Sensing Inhibitors

Quorum-sensing inhibition strategies, target bacterial communication pathways rather than biofilm structure. These systems are especially effective at preventing early-stage biofilm formation and reducing virulence. However, inhibition alone is insufficient for disrupting mature biofilms due to limited impact on established EPS architecture and bacterial protection mechanisms.

#### 2.3.5. Photocatalytic Systems

Photocatalytic nanocomposites generate ROS through light activation, enabling controlled antimicrobial effects with reduced off-target toxicity. Their advantages include tunable activation and compatibility with multifunctional designs. Nevertheless, their dependence on light penetration and oxygen availability restricts effectiveness in deep or poorly perfused wound tissues.

#### 2.3.6. Photothermal Systems

Photothermal systems, which generate localized heat upon irradiation (typically NIR), offer deeper tissue penetration and oxygen-independent activity. They are highly effective in disrupting EPS structure and killing bacteria rapidly. Nonetheless, achieving sufficient antimicrobial efficacy often requires high local temperatures or irradiation intensities, raising concerns about thermal damage to surrounding tissue.

#### 2.3.7. Stimuli-Responsive Nanocomposites

Stimuli-responsive nanocomposites represent a significant advancement over passive systems by enabling site-specific activation based on wound conditions (e.g., pH, enzymes, and ROS levels). These systems improve therapeutic precision, reduce systemic toxicity, and enhance biofilm targeting efficiency. However, their complexity introduces challenges in reproducibility, large-scale manufacturing, and regulatory approval.

#### 2.3.8. Multimodal Nanocomposites

By integrating complementary mechanisms, multimodal nanocomposites have the potential to outperform single-mechanism systems. These platforms can overcome microenvironmental constraints, target both structure and function, and contribute to reduce resistance development. However, their complexity introduces trade-offs in scalability, reproducibility, and regulatory approval, which remain major barriers to clinical translation.

## 3. Clinical Translation and Commercialized Examples of Nanocomposite Systems

Although most nanocomposite-based antimicrobial systems remain in preclinical development, a small number of FDA-cleared and clinically established wound-care products incorporating nanoscale materials have already reached routine medical use (Table 7). Currently, only a few wound-care products can be clearly classified as nanotechnology-based, and all are silver-nanostructures [192,193,194,195,196,197]. Their successful adoption demonstrates that nanocomposites can be translated into safe, scalable, and clinically effective wound therapies. These examples highlight the translational potential of next-generation nanocomposites.

## 4. Challenges and Limitations of Nanocomposites

### 4.1. Scientific Level

Biofilm microenvironments are dynamic and spatially heterogeneous, creating conditions that strongly contribute to wound chronicity [111]. Although nanocomposites frequently demonstrate strong antibacterial and anti-biofilm activity in in vitro systems, their performance in vivo often falls short of fully eradicating mature, multispecies biofilms. This can be due to the complex biofilm microenvironment and the dormancy of biofilm-embedded bacteria, which significantly reduces the nanocomposites’ efficacy [199].

Furthermore, balancing antimicrobial potency with host-cell biocompatibility remains a major scientific challenge. Metal-based nanocomposites (e.g., Ag, Cu, Au, ZnO, and Fe_3_O_4_) have been shown to exhibit dose-dependent cytotoxicity. At elevated concentrations, they can cause systemic absorption and organ accumulation, leading to genotoxicity, ROS-driven host-cell damage, inflammation, nephrotoxicity, and tissue damage. Furthermore, prolonged or uncontrolled release of metal ions may contribute to the emergence of drug-resistant bacteria. Hence, achieving selective toxicity that kills bacteria without damaging healthy tissue is challenging [1].

Therefore, to overcome these limitations, a deeper mechanistic understanding of biofilm resistance pathways, host-nanomaterial interactions, and the physicochemical parameters that dictate nanocomposite behavior are needed. To this end, when synthesizing metal and metal oxide nanoparticles, their size, shape, surface coating, kind of ligand, and charge need to be considered since these parameters dictate the level of toxicity produced by nanoparticles [200]. For example, nanoparticle size is one of the key factors affecting the antimicrobial properties of nanocomposites. Smaller nanoparticles expose more reactive surface per unit mass, increasing bacterial membrane contact, catalytic or redox activity, and the release of antimicrobial ions in metal-based nanoparticles. Nanoparticles less than 50 nm in size have demonstrated enhanced membrane disruption and intracellular damage. Furthermore, small-sized metal and metal-oxide-based nanocomposites have been shown to accelerate ion release, the generation of ROS, and higher biofilm diffusion, while larger particles ~100–200 nm remain in the biofilm surface causing reduced efficacy [201,202]. On the other hand, when nanoparticle size is too small, they can cause higher cytotoxicity to human cells, can aggregate faster when smaller than 10 nm, and produce an uncontrolled ion release [203]. Therefore, further research is required to elucidate the mechanisms underlying biofilm resistance to current therapies and to inform the design of optimized nanocomposite systems [1].

#### Immunogenicity

Nanocomposites used in chronic wound treatment, such as silver, gold, and graphene oxide-based nanocomposites, can exhibit immunogenicity due to their high surface energy and ability to trigger macrophage polarization (from M1 to M2). While designed to be biocompatible, these nanomaterials can induce immune responses that, if uncontrolled, may hinder rather than aid healing [204]. Additionally, nanoparticle-based systems have enhanced reactivity in vitro and in vivo due to increased surface area-to-volume ratios and interactions with biological components. These interactions cannot lead to adverse reactions such as hypersensitivity, depending on the system’s physicochemical properties [99,205].

### 4.2. Technical Level

#### Manufacturing and Reproducibility

Another challenge in the clinical translation of nanocomposite systems or nanomaterials is the ability to scale up production with accuracy. Maintaining precise shape, size, and uniformity at large volumes is difficult, making it hard to have batch-to-batch reproducibility. Furthermore, the environmental impact of scaling-up nanomaterials, including increased chemical waste and energy consumption, needs to be taken into account [206].

### 4.3. Translational Level

One major hurdle in evaluating the efficacy of nanocomposites against biofilms in chronic wounds is that many studies remain at the preclinical level, relying on in vitro assays, ex vivo wound models, and small animal models experiments. As a result, clinical safety, optimal dosing, regulatory pathways, large-scale manufacturing feasibility, and long-term outcomes have not yet been established. Most in vivo studies presented in this review use mice and rats, which present fundamental biological and translational limitations [207]. Rodent skin heals primarily through rapid wound contraction mediated by the panniculus carnosus muscle; in comparison, human wounds heal through the formation of granulation tissue, re-epithelization, and extracellular matrix remodeling.

Moreover, human chronic wounds arise from complex, multifactorial conditions such as vascular insufficiency, neuropathy, diabetes, and aging. Reviews of chronic wound models consistently conclude that no small-animal model captures the biochemical environment, chronicity, prolonged inflammation, impaired perfusion, or polymicrobial characteristic of human chronic wounds, creating a translational gap for advanced therapeutics [13,207].

Another major limitation is the absence of validated polymicrobial biofilm animal models that reflect the microbial ecosystem of chronic wounds. Most studies used only one or two bacterial strains, despite evidence that chronic wounds are polymicrobial, and that interactions between species increase bacterial resistance [29,109,208,209]. Consequently, many preclinical findings cannot be assumed to translate directly to human pathology [13,29,109,207,208,209].

To bridge this gap, there is an urgent need to develop large-animal models that accurately replicate human wound biology. Porcine skin, which closely resembles human skin in structure, immune response, and healing dynamics [210], represents a promising platform for establishing chronic wound and polymicrobial biofilm models that can better support the clinical translation of nanocomposite-based techniques.

### 4.4. Regulatory and Commercial Levels

Regulatory agencies have begun to address nanomedicine, but detailed frameworks for evaluating nanocomposite wound therapies are still evolving. Moreover, previous animal studies and clinical trials have established that it is challenging to validate nano-based systems; there are no FDA- or EMU-established guidelines to validate their safety and long-term stability, making it difficult to establish the toxicity level and understand their overall effect systematically [204]. Commercial viability requires strict quality control over nanoparticle size, distribution, surface chemistry, and matrix integration, as well as standardized characterization methods that are more acceptable to clinicians. The lack of universally accepted standards for nanocomposite wound products complicates cross-study comparisons, regulatory review, and procurement decisions in healthcare systems [211].

## 5. Future Perspective

To better understand the overall effects of nanocomposite systems when used to eradicate biofilms in chronic wounds, more robust guidelines to test for long-term toxicity stability testing, and reproducibility specifically for nanomaterials should be established. Furthermore, experimental designs should incorporate analysis to better understand the mechanism of action in eradicating biofilms and promoting wound healing and will help in understanding the long-term toxicity caused by nanocomposites, which will promote the development of systems that target and deliver individualized treatment.

## 6. Conclusions

This review highlights how biofilms act as precursors to environmental changes within chronic wounds. We explore how nanocomposites can be engineered to combat these biofilms and support wound healing. Multimodal nanocomposite systems leverage effects such as photothermal, photocatalytic, and quorum-sensing inhibition to disrupt biofilms, eradicate bacteria, and reduce side effects. These integrated systems not only overcome bacterial resistance more effectively but also promote wound healing by modulating the wound microenvironment. By harnessing the complementary strengths of different modalities, synergistic nanocomposites provide superior therapeutic outcomes for complex chronic wounds compared to any single-modality approach.

In conclusion, multimodal nanocomposites can serve as alternatives to target specific mechanisms of antibiotic resistance and various stages of biofilm formation [212]. Furthermore, given their multifunctional features, they can be an effective tool to address the limitations of current treatments and create systems with synergistic effects that not only disrupt biofilms and eradicate bacteria but also simultaneously promote wound healing by reducing inflammation, promoting re-epithelialization, stimulating angiogenesis, and restoring cell functionality. However, to further translate nanocomposite systems for the treatment of biofilms in chronic wounds, it is fundamental for scientists, clinicians, and researchers to better collaborate and share their results to create an interdisciplinary research consortium database, enabling a multi-tiered approach that addresses the complexity of the biofilms’ nature and the patient’s overall health.

## Figures and Tables

**Table 1:** Comparison of acute wound phases vs. chronic phases.

**Acute Wound Healing Process**			
**Hemostasis**	**Inflammation**	**Proliferation**	**Maturation and Remodeling Phase**
-Vasoconstriction of blood vessels -Activation, adhesion, and aggregation of platelets to stop bleeding -Intrinsic and extrinsic coagulation pathways are activated -Vasodilation -Stabilize fibrin and platelet clot	-Neutrophils infiltration -Chemotaxis -Increased vascular permeability -Debris and bacteria removal -Occurs within the first 24 hrs. -Lasts up to 2 weeks	-Replacement of provisional fibrin matrix -Formation of granular tissue -Fibroblast migration -Collagen and extracellular matrix synthesis -Re-epithelialization -Neovascularization	-Collagen remodeling -Vascular maturation -Wound matures and reaches maximum strength
**Chronic Wound Healing Process**			
**Hemostasis**	**Inflammation**	**Proliferation** **Maturation and Remodeling Phase**	
Same as in acute wounds	-Chronic inflammatory state -Increased number of neutrophils, macrophages, and lymphocytes -Increased levels of proteases and reduced numbers of protease inhibitors -Reactive oxygen species (ROS) elevated levels -Prolonged inflammation that lasts months/years	-Deficiency of TGF-β -Fibroblast senescence -High levels of matrix MMPs -Excess proteolysis -Reduced epithelization -Impaired angiogenesis -Degradation of ECM proteins and growth factors	

**Table 2:** Types of chronic wounds and their risk factors.

Type of Chronic Wounds	Extrinsic Risk Factors	Intrinsic Risk Factors	References
Pressure Ulcers	Long-term immobilization Smoking	Diabetes Malnutrition	[15]
Diabetic Ulcers	Unperceived trauma with sensory neuropathy Foot deformity Ill-fitting shoes Mechanical stress	Hyperglycemia Oxidative stress Peripheral vascular disease Immunopathy Neuropathy	[16,17]
Venous Ulcers	Obesity Injury Older age Smoking	Chronic venous insufficiency Damaged valves Varicose veins Hypertension Osteoarthritis	[18,19,20]
Arterial Ulcers	Older age Smoking Obesity Decreased activity	Atherosclerosis Diabetes Thromboangiitis	[20]

**Table 3:** Bacterial species commonly present in chronic wounds.

Name	Characteristics	Chronic Wounds Biofilms	References
Methicillin-resistant *S. aureus* (MRSA)	▪ Gram-positive ▪ Facultative anaerobic ▪ Coccal-shaped	▪ One of the four most prevalent bacterial species ▪ Causes enhanced virulence ▪ Provides a protective barrier against antibiotics ▪ Increased risk or recurrence ▪ *S. aureus* biofilms are present at the wound surface	[29,37,38]
*Escherichia coli* (*E. coli*)	▪ Gram-negative ▪ Rod-shaped ▪ 1–3 × 0.4–0.7 μm in size ▪ Facultative anaerobe	▪ Can contribute to delaying healing ▪ Can enter the blood and cause sepsis	[39]
*P. aeruginosa*	▪ Gram-negative ▪ Facultative anaerobe ▪ 1.5–3 × 0.5–0.8 μm ▪ Motile-single flagellum ▪ Rod-shaped	▪ 51.7% representation in venous ulcers ▪ *P. aeruginosa* biofilms deeper within the wound	[29]
*Finegoldia magna* (*F. magna*)	▪ Gram-positive ▪ Anaerobic coccus	▪ Acts as an opportunistic pathogen ▪ Can form biofilms ▪ Induces inflammation	[40]

**Table 5:** Antimicrobial and biofilm targeting nanocomposite mechanisms.

Mechanism	Biological Action	Nanocomposite Strategies	Advantages	Limitations	Key Design Parameters	Ref.
ROS generating	-Oxidative damage to lipids, proteins, and DNA -Biofilm matrix oxidation -Metabolic collapse	-Metal, metal-oxide based ROS generators (Ag^+^, Cu^2+^, and Fe^2+^) -Photocatalytic nanoparticles (TiO_2_, ZnO, and g-C_3_N_4_)	-Broad spectrum antimicrobial activity -Reduced resistance potential -Synergy with chronic wound environment -Deep biofilm penetration	-Dose-dependent cytotoxicity -Uncontrolled production ROS -Short ROS lifetime -Inactivation tissue inhibitors of MMPs promoting high proteolytic microenvironment and inhibiting wound healing -Reduced efficiency in hypoxic wound environments	-Bandgap engineering to optimize light absorption -Balancing ROS generation -Surface charge and hydrophilicity to ensure penetration into EPS	[64,106,107,108]
EPS matrix dispersal/ degradation	-Polysaccharide degradation -Protein cleavage -Enzyme-mimetic activity (nanozymes)	-Enzyme-loaded nanocomposites (DNase, protease, and dispersing B) -Nanozymes (peroxidase-like, oxidase-like, and DNase-like)	-Improved penetration into biofilms -Reduces biofilm mechanical stability -Destruction EPS matrix allows immune cells to access, engulf, and kill bacteria	-EPS composition varies by species -Enzyme instability -Potential off-target degradation -Slow action	-Choice EPS-targeting agent according to bacteria species -Size and charge NPs (smaller, cationic NPs penetrate EPS more effectively) -Synergy other mechanism to kill bacteria	[109]
Contact-killing/membrane disruption	-Membrane rupture -Lipid peroxidation -Pore formation -Leakage of intracellular contents	-Cationic polymer-metal nanocomposites -Sharp-edged nanostructures -Surface-functionalized nanoparticles -Photothermal nanocomposites	-Immediate bactericidal action -Low risk of resistance -Effective in low-oxygen or hypoxic wounds -Works well against dormant bacterial cells	-Potential cytotoxicity to host cells -Limited penetration -Aggregation issues -Short-range mechanism	-Surface charge tuning (mildly cationic surfaces enhance bacterial binding) -Surface functionalized with antimicrobial peptides, quaternary ammonium groups	[110,111]
Quorum sensing inhibition/ metabolic interference	-Binding or degrading autoinducers -Blocking receptor-ligand interactions -Inhibiting autoinducer synthesis enzymes -Inhibition of energy production	-Silver, gold, or zinc oxide nanoparticles	-Prevent biofilm formation at early stages -Reduces EPS production -Lowers virulence without strong selective pressure -Synergizes with ROS, antibiotics, and EPS-degrading enzymes	-Species-specific -Slower action -Harder to disrupt mature biofilms -Requires sustained presence to block signaling	-Targeting specific autoinducers (AHLs, AI-2, and PQS) -Controlled release to maintain inhibitory concentrations -Nanocarrier selection for stability in wound fluid -Co-delivery with ROS or antibiotics for synergy	[112]

**Table 6:** Nanocomposites to target biofilm in chronic wounds.

Material	Antimicrobial Mechanism	Model	Results	Ref.
**Reactive Oxygen Species (ROS)–Generating Nanocomposites**				
Cationized silica ceria nanocomposite	Induction of oxidative stress by generation of ROS and inhibition QS by acting as redox-active catalyst	**In vitro** *E. coli* and *S. aureus* biofilm	99.9% inhibition against *S. aureus* and 81% against *E. coli*. 80% inhibition biofilm, 77% disruption biofilm+	[120]
Polydopamine NPs/gelatin oxidized dextran nanocomposite (Gel-oDex-PDA-PHMB)	Production ROS to induce oxidative stress	**In vitro** *S. aureus*, *E. coli*, and MRSA biofilm **In vivo** Streptozotocin induced diabetic Sprague-Dawley *S. aureus* wound infection model	**In vitro** 99% reduction in bacterial viability **In vivo** 90% bacterial viability reduction	[104]
**(a) Metal, metal-oxide-based**				
Dopamine-modified barium titrate NPs/poly (vinylidene fluoridetrifluoroethylene) (P(VDF-TrFE)) matrix nanocomposite membrane	Membrane disruption through surface electrical potential/increment intracellular ROS	**In vitro** *S. aureus* biofilm formation **In vivo** Full-thickness cutaneous defect mouse model with *S. aureus* infection (24 hrs.)	**In vitro** Increased death rates of *S. aureus* biofilm bacteria **In vivo** Infection inhibition and wound healing acceleration	[176]
Polyethyleneimine surface-modified silver-selenium nanocomposites (ASP NCs)	ROS burst through electrostatic interaction	**In vitro** *S. aureus* and *E. coli* **In vivo** *E. coli* and *S. aureus* mice infected model	**In vitro** Inhibition of biofilm formation **In vivo** 80% reduction in wound size	[177]
Titanium dioxide nano-formulation	Metal ion release and ROS production	**In vitro** *S. aureus* and *E. coli* antibiofilm assay **In vivo** *S. aureus* infected ICR mice model	**In vitro** Reduction biofilm formation by 99% **In vivo** No significant difference in number of bacteria reduced in treatment group compared with negative or positive control, but treatment group had the highest early wound healing score (EHS) of 9.81 ± 0.21	[178]
Zinc copper oxide (ZnCuO_2_) nanoparticles/borax bacterial cellulose nanocomposite	Metal ions release	**In vitro** *Listeria innocua*, *S. aureus*, *P. aeruginosa, E. coli*, *Streptococcus parasanguinis*, and *Candida albicans* (*C. albicans*) biofilms	36.09–90.43% biofilm eradication	[179]
Collagen-based Ag-hydrogel nanocomposite	Metal ions release	**In vitro** MRSA and *P. aeruginosa* **In vivo** MRSA-infected Ti disc implants in Sprague Dawley rats	**In vitro** 4.56 ± 0.25 log_10_ reduction (LR) MRSA 4.20 ± 0.33 LR *P. aeruginosa* **In vivo** Greatest reduction (6.43 ± 0.38 LR) at concentration of 600 ppm Ag	[180]
Neodymium (Nd) and Europium (Eu) High-entropy iron-oxide nanozymes (C-ZnFeO@Eu and C-ZnFeO@Nd)	ROS generation	**In vitro** MRSA and *P. aeruginosa* **In vivo** MRSA-infected BALB/c mice	**In vitro** C-ZnFeO@Eu 80% inhibition against MRSA C-ZnFeO@Nd 98% inhibition against MRSA **In vivo** 90.2% wound closure at day 8 and 100% at day 10	[181]
**(b) Photocatalytic**				
Graphene oxide (GO)/copper (Cu) doped zinc oxide (ZnO) nanocomposite (GO/Zn (Cu)O)	Photo-induced ROS production and bacterial inhibition	**In vitro** *S. aureus*, *Enterococcus faecium* (*E. faecium*), *E. coli*, *S. typhi, Shigella flexneri* (*S. flexneri*), and *P. aeruginosa* biofilms	72% reduction of EPS content	[128]
Vanadium-nitrogen-doped TiO_2_ nanoparticles with graphene oxide nanocomposite (GO/TiO_2_(V-N))	ROS and photothermic generating superoxide and hydroxyl radicals and producing bacteria lysis	**In vitro** *E. coli*, *P. aeruginosa*, *S. aureus*, and *Enterococcus* (*E. faecalis*) biofilms (24 hrs.)	Biofilm formation inhibition by 90% EPS reduction: *E. coli:* ~69 ± 0.6% *P. aeruginosa:* ~64 ± 0.4% *S. aureus:* 57 ± 0.7% *E. faecalis:* 55 ± 0.5% Biofilm disruption: *E. coli:* ~56% ± 0.6% *P. aeruginosa:* ~49% ± 0.7% *S. aureus:* 43% ± 0.7% *E.faecalis:* 36% ± 0.6%	[127]
MgO/Cu-PCL MgO/Ag-PCL nanofibers	ROS generation	**In vitro** *S. aureus* and *E. coli* **In vivo** *S. aureus*-infected ICR mice	**In vitro** 99% *S. aureus* antimicrobial activity 82% *E. coli* **In vivo** 90% wound closure after 11 days	[182]
GO/Zn (Cu) O nanocomposite	ROS generation	**In vitro** *S. aureus, E. faecium, E. coli, S. flexneri, S. typhi,* and *P. aeruginosa*	95% antibiofilm activity Biofilm disruption *S. aureus*~47.8 ± 2.39% and 51.1 ± 2.55%, *E. faecium*~44.4 ± 2.22% and 50 ± 2.49%, *E. coli*~57.2 ± 2.86% and 66.5 ± 3.32%, *S. typhi*~48.1 ± 2.40% and 53 ± 2.64%, *S. flexneri*~53.2 ± 2.66% and 59.7 ± 2.98% *P. aeruginosa*~59.5 ± 2.97% and 68.3 ± 3.41%	[128]
**EPS Dispersal/Degradation**				
**(a) Enzymatic-functionalized**				
Nanocellulose gel containing niosomal serratiopeptidase (NioSTP-BNC)	Proteolytic biofilm dispersal	**In vitro** *S. aureus* and *P. aeruginosa* biofilms	53.38% biofilm inhibition 1:1 ratio NioSTP-BNC gel against *P. aeruginosa* and 52.29% against *S. aureus*	[137]
Poly (lactic acid)-hyperbranched polyglycerol (PLA-HPG) based bioadhesives nanoparticles (BNPs) and chitosan (CS) network (SNO/BNP/Cs@Am-Cef)	Biofilm dispersal induced by NO, hydrolyzation of EPS polysaccharides by α Am, and cefepime bacterial lysis induced penicillin-binding proteins (PBPs)	**In vitro** MSSA and MRSA biofilm **In vivo** MRSA biofilm infected C57BL/6J mice	**In vitro** 96%-MSSA and 90%-MRSA biofilm dispersal efficiency and 93% viability reduction MRSA **In vivo** Reduction of bacteria from ~350 × 10^5^ CFU to ~2 × 10^5^ CFU	[183]
Chitosan gel silver sulfadiazine nanoparticles (SLNs) deoxyribonuclease-I (DNase-I) nanocomposite	eDNA hydrolysis and electrostatic interactions	**In vitro** 48 hrs. *P. aeruginosa* biofilm **In vivo** Wistar albino rat burn wound model	**In vitro** Nanocomposite obtained 96.8% biofilm inhibition in comparison with Silver Sulfadiazine/DNase-I which only obtained 82.9% **In vivo** 100% wound closure after 21 days	[141]
**Contact Killing and Membrane Disruption**				
**(a) Electrostatic interaction**				
Octenisept (OCT) and Prontosan (loaded bacterial nanocellulose (BNC)	OCT-Cationic surfactant attaches and disrupts bacterial cell membrane, depolarizing membrane and causing cytoplasm leakage Prontosan-Binds cell membrane and causes structural disruption, softens and detaches biofilm	**In vitro** 24 hrs. *S. aureus* and *C. albicans* biofilms. 48 hrs. *P. aeruginosa* biofilm **In vivo** Porcine excisional wound model	**In vitro** *P. aeruginosa.:* 2.7 LR (Prontosan) and 2.6 LR (Octenisept) *S. aureus*.: complete eradication after 24 hrs. (Octenisept) **In vivo** Wound closure > 75%	[184]
Chitosan/Silver nanocomposite	Exopolysaccharide degradation	**In vitro** *S. aureus* biofilm	96% biofilm degradation after 24 hrs.	[185]
Poly (2-hydroxyethylmethacrylate)-chitosan hydrogels loaded cerium oxide nanocomposites (PHEM-CS/CeONPs)	Chitosan electrostatic disruption of bacterial cell membranes and metal chelation	**In vitro** *E. coli* and *S. aureus* biofilms **In vivo** Sprague Dawley rats no biofilm	**In vitro** ~90% *S. aureus* biofilm reduction ~100% *E. coli* biofilm reduction **In vivo** 98.5 ± 4.95% wound closure achieved	[103]
LL37-loaded chitosan/ZnO nanocomposite (CS/ZnO-NCs)	Release of LL37 peptide-electrostatic disruption of bacterial membrane, causing pore formation and lysis and EPS, inhibition QS.	**In vitro** MRSA biofilm	81% biofilm formation inhibition 6 LR MRSA	[186]
Polymer-silver chloride nanocomposite (QAm1Agn)	Bacterial membrane permeabilization and depolarization	**In vitro** *E. coli*, *P. aeruginosa* biofilm (48 hrs.), and polymicrobial biofilm (*C. albicans* and MRSA (24 h) **In vivo** Neutropenic mice dorsal skin *P. aeruginosa* biofilm model	**In vitro** *E. coli* and *P. aeruginosa:* At 32 and 64 μg/mL, 3.5 log and 5.6 log reduction, at 128 μg/mL, complete eradication was observed Polymicrobial biofilm: 2.1 log reduction (256 μg/mL) **In vivo** At 25 mg/kg > 99% killing of bacteria, at 50 mg/kg, 3.5 LR (>99.9%)	[119]
Hyaluronic acid/LAP@W379 antimicrobial peptide nanoparticles injectable nanocomposite (HDL-1@W379)	W379– Activating MEK/ERK signaling pathway to produce bacteria cell death	**In vitro** MRSA biofilm (72 hrs.) **In vivo** MRSA-infected high-fat diet, diabetic KM mice model	**In vitro** Biofilm dispersal and CFU reduction by ~99.95% **In vivo** Reduction of MRSA CFU from 9 to >1	[187]
**(b) Photothermal (PTT)**				
Pectinase (Pec) molybdenum disulfide nanosheets (Pec@PLL-MoS_2_)	Enzymatic degradation of EPS by hydrolysis. degradation and biofilm. Photothermal activation of Pec	**In vitro** Mature biofilms of *S. aureus* and *E. coli* **In vivo** Male Sprague-Dawley (SD) rats infected with polymicrobial biofilm (48 hrs.)	**In vitro** 94.9% of *S. aureus* and 99.9% of *E. coli* eliminated within 5 min. of irradiation **In vivo** 0.1% bacterial survivor rate after 3 days. Results showed complete wound closure after 9 days	[188]
Polydopamine/cooper peroxide/ICG nanocarrier (PDA/CP/ICG)	Thermal decomposition of CP into O_2_ Oxidative stress to kill bacteria by lipid peroxidation and damage to proteins and DNA	**In vitro** *S. aureus* biofilm **In vivo** Balb/c mice wound infection model with *S. aureus*	**In vitro** 82.5% biofilm elimination with PDA/CP/ICG + NIR vs. 74.1% without NIR **In vivo** ~13.5% residual wound area after 5 days indicating near-complete biofilm eradication	[189]
Kanamycin-derived carbon-nanodots (KCDs) and nitric oxide donors (BNN6) loaded into dendritic silica-coated three-layer core-shell conversion materials (USKB)	Disruption antioxidant system of MRSA inhibiting bacterial ATP synthesis Triple ROS (O_2_^−^, NO which produce the generation of ONOO–)	**In vitro** MRSA biofilm (48 hrs.) **In vivo** Streptozotocin induced diabetic MRSA-infected Kumming mice model **Irradiation:** Laser (980 nm, 10 min.)	**In vitro** ~95.9% biofilm clearance **In vivo** 99.9% bacterial reduction	[153]
Pt-decorated gold nanoparticles with sodium nitroprusside (SNP)-loading porphyrin metal organic framework (PCN)	Gas-photodynamic-photothermal killing by producing hyperthermia, ROS generation and trigger NO release	**In vitro** *E. coli*, MRSA, *S. aureus*, *S. epidermidis*, *S. typhi*, *K. pneumoniae*, and *P. aeruginosa* **In vivo** MRSA biofilm-infected mice model (72 hrs.) **Irradiation:** Laser (808 nm, 1.0 W/cm^2^, 10 min.)	**In vitro** 99% bacterial inhibition within 10 min. **In vivo** 99.9% reduction in bacterial burden on wounds	[154]
**Quorum-Sensing Inhibition (QS)**				
AIE/LA@HMONs-PyB	QS disruption ROS induced oxidative stress. Disruption of metabolic pathways (glycerol lipid and histidine)	**In vitro** MRSA biofilm **In vivo**-Streptozotocin induced diabetic mice model with MRSA biofilm **Irradiation:** Laser (660 nm)	**In vitro** Antimicrobial efficiency of 99.99% **In vivo** ~2.9 LR MRSA	[190]
Carvacrol incorporated into cross-linked poly (oxanorbornenimide) polymers (PONIs) nanocomposite	QS disruption, adhesion reduction and EPS degradation	**In vitro** *P. aeruginosa* and *S. aureus* biofilms	Eradication of 90% bacteria in biofilms at concentrations from 2–43 *v*/*v*%	[161]
**Multimodal-Synergistic Mechanism**				
Chitosan-indocyanine green/luteolin nanocomposite (ICG/LUT-CS)	Photothermal therapy and quorum-sensing inhibition	**In vitro** *S. aureus* biofilm **In vivo** *S. aureus*-infected BALB/c mice model	**In vitro** Antimicrobial almost 99.9% Biofilm inhibition 99.9% Eradication 64.78% after 24 hrs. **In vivo** Day 8 ~89% wound closure and almost all residual live bacteria were eradicated reducing OD_600_ to 0.10 ± 0.002	[175]
Iron-doped Bi2O2S (Fe-Bi2O2-XS, Fe-BOS) and adipic acid dihydrazide-modified CS/oxidized HA (Fe-BOS@C/H Gel)	Photothermal and ROS generation	**In vitro** MRSA biofilm **In vivo** MRSA-infected mice model	**In vitro** >90% elimination rate **In vivo** Antibacterial rate of 94.81% ± 1.32% Wound-closure rates of 97.29% ± 0.31% on day 8	[191]
Selenium-tellurium doped copper oxide nanoparticles (SeTe-CuO NPs)	Hyperthermia and release of metal ions that enhance ROS production	**In vitro** *E. coli* and *S. aureus* biofilms **In vivo** *E. coli*-infected wound BALBc/mice model	**In vitro** 80% inhibition *E. coli* biofilm and 70% *S. aureus* 65% *E. coli* biofilm mass reduction, 62% *S. aureus* biofilm mass reduction **In vivo** 100% bacterial eradication after 12 days and 100% wound healing	[174]

**Table 7:** Commercial nanoparticle-based dressings used in chronic wounds.

Commercial Nanoparticle-Based Dressings Used in Chronic Wounds					
Product	Company	Nanomaterial	Mechanism	Clinical Indication	Ref.
Anticoat^TM^	Smith & Nephew	Nanocrystalline silver (10–20 nm)	Sustained Ag^+^ release Membrane disruptions Enzyme activation	Chronic wounds Burns	[192,193,194]
NanoSALV (NanoSALV Inc.)	Nanosalv Inc	Nanoparticle gel	Catalytic antimicrobial activity Inflammation reduction Enhancement tissue repair	Diabetic foot ulcers, Venous leg ulcers Pressure ulcers Arterial ulcers Surgical wounds Burns	ICH-GCP. NanoSALV Clinical Trial NCT05619237.
Aquacel Ag/Ag^+^ Extra (Convatec)	ConvaTec	Silver nanoparticles dispersed in hydrofiber	Ag^+^ release Moisture-activated Broad-spectrum antimicrobial	Diabetic foot ulcers Venous leg ulcers Pressure ulcers Burns	[195]
Algidex Ag	Nobel Biocare/Cura Metix	Silver nanoparticles foam/paste	Large-surface-area antimicrobial contact	Pressure ulcers Venous stasis ulcers Diabetic ulcers Partial-thickness burns	[198]
Mepilex Ag	Molnlycke	Silver nanoparticles in polyurethane foam	Exudate control Silver-ion mediated bacteria killing	Chronic ulcers Partial-thickness burns	[196]
Silverlon	Argentum Medical	Metallic silver plating with nano-scale surface features	Biofilm suppression Ag^+^ high-surface-area release	Chronic ulcers Surgical wounds Burns	[197]

## Data Availability

No new data were created or analyzed in this study. Data sharing is not applicable to this article.

## Abbreviations

The following abbreviations are used in this manuscript:

AMR	Antimicrobial resistance
AIs	Autoinducers
BL	Blue light
CFUs	Colony-forming units
DAMPs	Damaged-associated molecular patterns
DFU	Diabetic foot ulcers
ECM	Extracellular matrix
EGF	Epidermal growth factor
EPS	Extracellular polymeric substances
HIF-1α	Hypoxia-inducible factor-1 alpha
IL-10	Interleukin 10
LR	Log reduction
MMPs	Matrix metalloproteases
MRSA	Methicillin-resistant *S. aureus*
NAPDH	Nicotinamide adenine dinucleotide phosphate
NO	Nitric oxide
NPs	Nanoparticles
NPWT	Negative pressure wound therapy
PAMPS	Pathogen-associated molecular patterns
PDGFβ	Platelet-derived growth factor
PTAs	Photothermal agents
PTT	Photothermal therapy
PtO_2_	Partial pressure of oxygen
QS	Quorum sensing
ROS	Reactive oxygen species
TiO_2_	Titanium oxide
TGF-β1	Transforming growth factor beta-1
VEGF	Vascular endothelial growth factor
